# Supplementary material for: The First Whole Genome Sequence and Characterisation of Avian Nephritis Virus Genotype 3
Source: Viruses. 2021 Feb 3;13(2):235. doi: 10.3390/v13020235 (PMC7913312; doi:10.3390/v13020235)

Supplementary Figure S1. Intropro scan VF14-92-A2 ANV ORF1a  **(**MT585643)


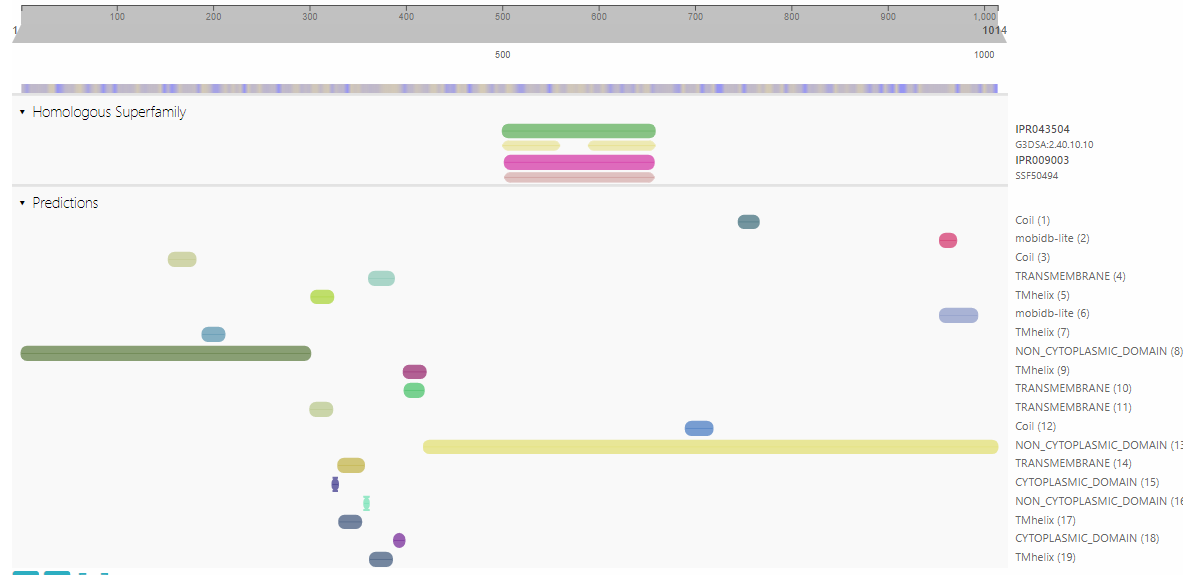


Supplementary Figure S2. Intropro scan VF14-92-A2 ANV ORF1b (MT585643)


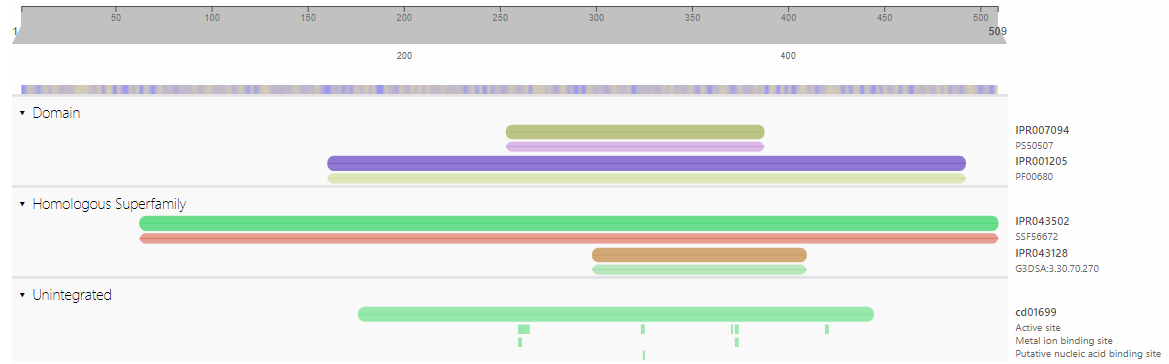

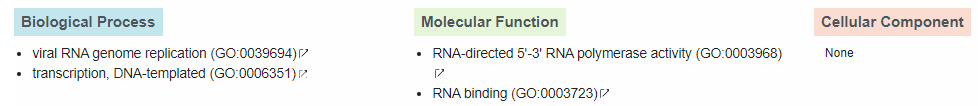


Supplementary Figure S3. Intropro scan VF14-92-A2 ANV ORF2 (MT585643)


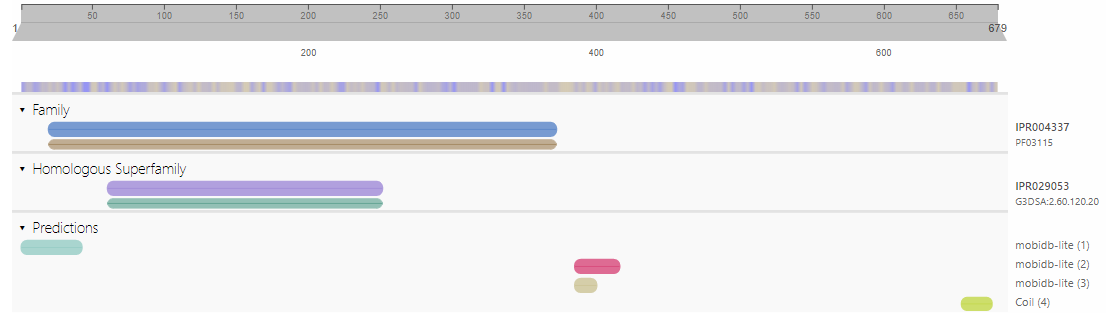


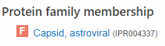


Supplementary Figure S4. MT585643 VF14-92-A NCBI Conserved Domains-Trypsin-like peptidase domain-containing protein (domain architecture ID 10595581) may function as a serine protease, catalysing the cleavage of peptide bonds in target proteins using serine as the nucleophilic amino acid


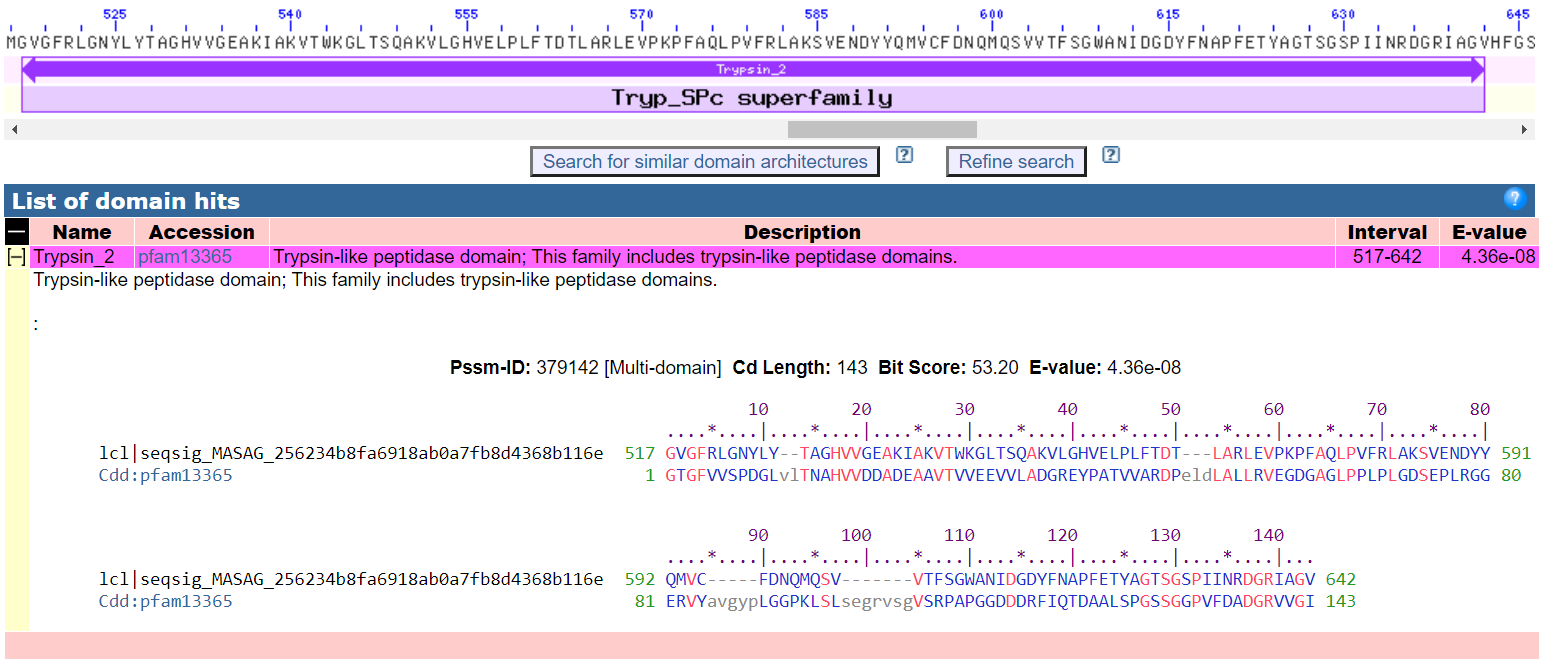


Supplementary Figure S5. Intropro scan of ORF1a VF16-03-164b (MT585644)


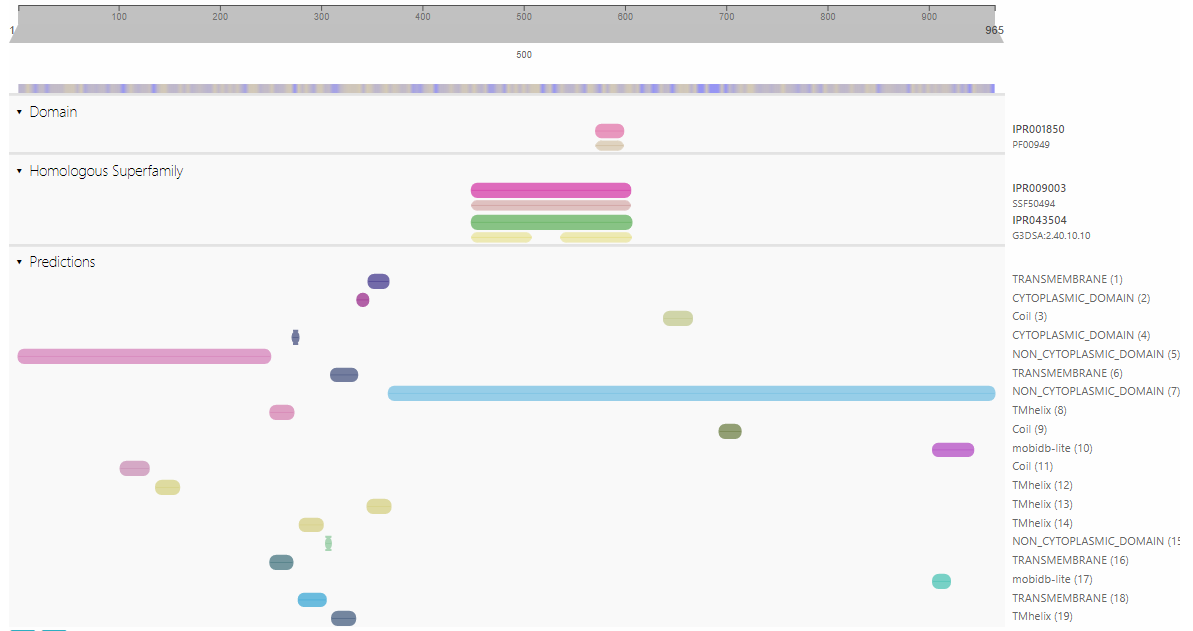


Supplementary Figure S6. Intropro scan of ORF1b VF16-03-164b 3 ORF1b (MT585644)


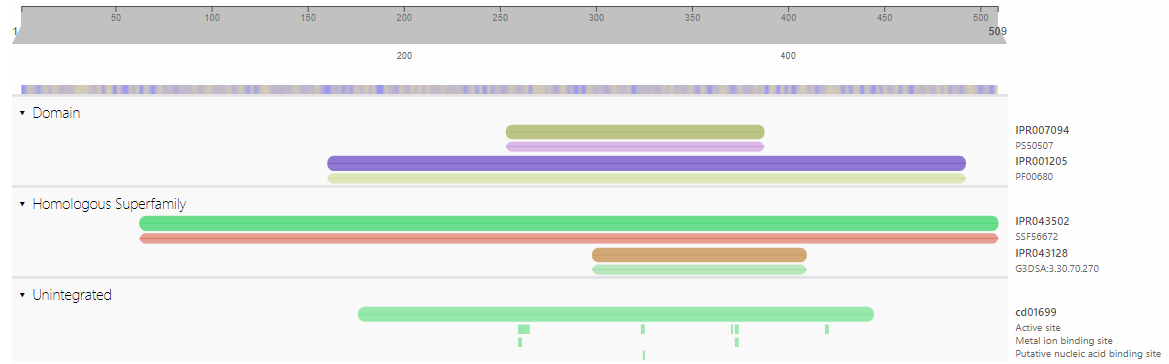


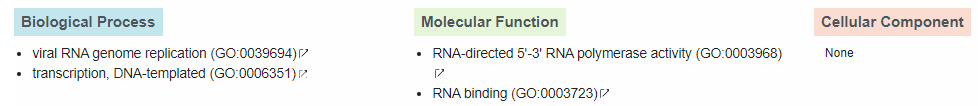


Supplementary Figure S7. Intropro scan of VF16-03-164b ANV-3 ORF2 (MT585644)


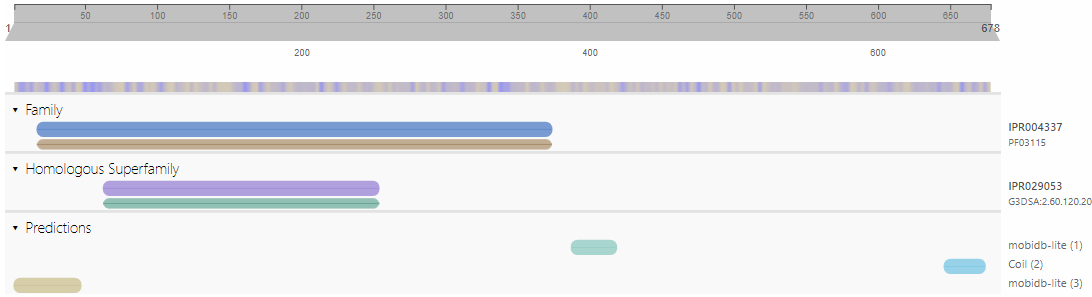

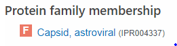

Supplement: Supplementary file 1 [file viruses-13-00235-s001.zip › Supplementary Figure S1 to S7 Introproscan.docx]
