# Supplementary material for: The First Whole Genome Sequence and Characterisation of Avian Nephritis Virus Genotype 3
Source: Viruses. 2021 Feb 3;13(2):235. doi: 10.3390/v13020235 (PMC7913312; doi:10.3390/v13020235)
Supplement: Supplementary file 1 [file viruses-13-00235-s001.zip › Supplementary Figure S8. 3UTR s2m stemloop.pptx]

## Slide 1
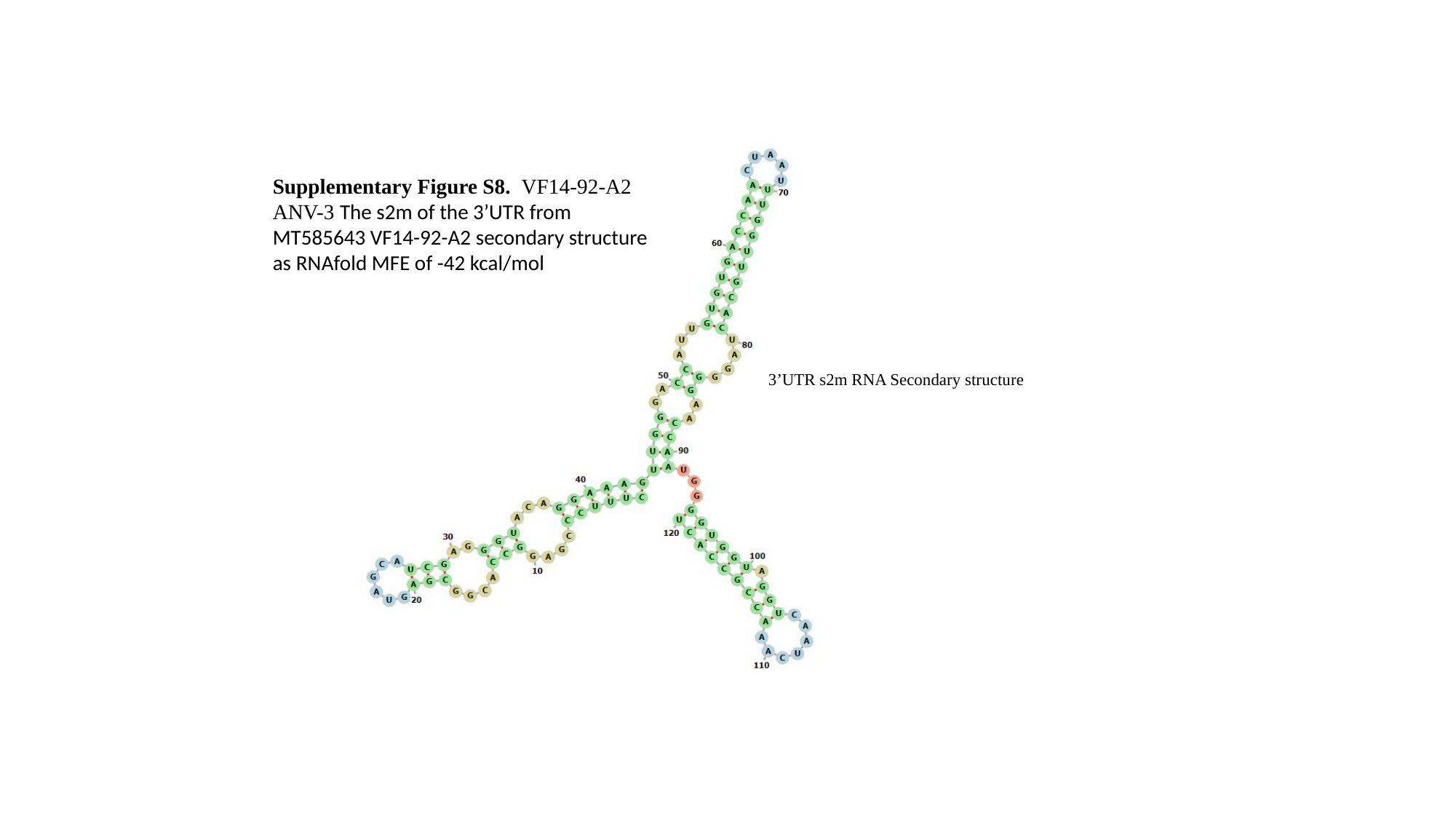

Supplementary Figure S8. VF14-92-A2 ANV-3 The s2m of the 3’UTR from MT585643 VF14-92-A2 secondary structure as RNAfold MFE of -42 kcal/mol
3’UTR s2m RNA Secondary structure
