# Supplementary material for: The First Whole Genome Sequence and Characterisation of Avian Nephritis Virus Genotype 3
Source: Viruses. 2021 Feb 3;13(2):235. doi: 10.3390/v13020235 (PMC7913312; doi:10.3390/v13020235)
Supplement: Supplementary file 1 [file viruses-13-00235-s001.zip › Supplementary Figure S9a&b RDP.docx]

**Supplementary Figure S9a. VF14-92-A2 ORF2 RDP**

**Supplementary Figure S9b. VF16-03-164b ORF2 RDP**

The nucleotide position breakpoints for VF14-92-A2 was located at 1374-2258 and VF16-03-164b positions as 1298–2252. The VF14-92-A2 potential major parent was KU711052 (ANV-8), and potential minor parent KM985698 VIC-3c (ANV-1), and VF16-02-164b inferred unknown potential parent HO086767 (ANV-1) and minor parent KM985694 NSW-4a (ANV-1).
