# Supplementary material for: The First Whole Genome Sequence and Characterisation of Avian Nephritis Virus Genotype 3
Source: Viruses. 2021 Feb 3;13(2):235. doi: 10.3390/v13020235 (PMC7913312; doi:10.3390/v13020235)
Supplement: Supplementary file 1 [file viruses-13-00235-s001.zip › Supplementary Table S3 RDP4 p-values.docx]

**Supplementary Table S3. Putative Recombination Events ANV (RDP4 software *P-*value)**

| Sample Name | VF14-92-A2 | VF16-03-164B | |
| --- | --- | --- | --- |
| Breakpoints  (nucleotide position) | **1374 - 2258** | | **1298 - 2252** |
| Programs Average *p-*value | | | |
| RDP | **9.58 x 10^-3^** | | **1.031 x 10^-10^** |
| GENECONV | **9.005 x 10^-1^** | | **1.830 x 10^-5^** |
| BootScan | **3.854 x 10^-2^** | | **1.881 x 10^-9^** |
| MaxChi | **2.053x 10^-5^** | | **1.558 x 10^-10^** |
| SiScan | **1.001 x 10^-16^** | | **5.135 x 10^-19^** |
| Phylpro | **3.275 x 10^-4^** | | **3.885 x 10^-20^** |
| LARD | **1.327 x 10^-6^** | | **5.810 x 10^-31^** |
| 3Seq | **3.275 x 10^-4^** | | **3.885 x 10^-20^** |
